# Neglected players: Tumor associated neutrophils involvement in chronic lymphocytic leukemia progression

**DOI:** 10.18632/oncotarget.26716

**Published:** 2019-03-08

**Authors:** Enrique Podaza, Denise Risnik

**Affiliations:** Laboratorio de Inmunología Oncológica, IMEX-CONICET, Ciudad de Buenos Aires, Argentina

**Keywords:** chronic lymphocytic leukemia, tumor associated neutrophils, IL-8

The old paradigm that establishes that neutrophils are just short half-life cells committed to kill bacteria *in situ* at early stages of infection is changing. Nowadays, there are plenty of reports that focus on neutrophils participation in tumor microenvironment giving rise to the so-called Tumor Associated Neutrophils (TANs). TANs appear to be very sensitive to microenvironmental signals increasing their lifespan and displaying remarkable phenotype plasticity and functionality. Even though defining a unique phenotype to classify TANs populations in cancer patients is complicated due to the lack of appropriate surface markers [1], their role as tumor promoters is supported by clinical observations that associate a higher neutrophil infiltrate at the tumor [2] and/ or a high neutrophil to lymphocyte ratio with poor outcome [3]. Most of the reports regarding TANs biology, both in murine models and patients, are in the context of solid tumors [1]. In 2016, our group described for the first time that neutrophils from Chronic Lymphocytic Leukemia (CLL) patients were prone to release extracellular traps (NETs) and that these structures promote leukemic cells activation and survival [4]. These findings encourage us to think about the possibility that leukemic cells could drive TANs differentiation in CLL. In 2018, we go further and reported that leukemic cells promote neutrophil survival through G/GM-CSF release and their IL-10/TGF-β- driven reprogramming into CD16highCD62Ldim subset, which is capable of significantly suppress T-cell functions (proliferation and INFγ production) [5]. We aim directly at this subset, described in head and neck squamous cell carcinoma patients [6], given that these neutrophils exhibit a higher capacity to release NETs and are present in peripheral blood. We observed not only that leukemic cells were able to reprogram circulating neutrophils into this subset *in vitro*, but also that the proportion of these TANs was increased in CLL-patients.

A common feature of our work and that from Millrud et al is IL-8 involvement. We described that higher IL-8 plasmatic levels in CLL-patients were responsible of the increased NETs formation observed *in vitro* upon stimulation while Millrud reported that CD16highCD62Ldim neutrophils are sensitized to respond to IL-8 migrating rapidly to tumor tissues.

It is known that higher plasmatic levels of IL-8 in CLL-patients [7] are related with poor prognosis but as we previously described [8] it is not a consequence of a direct action of this chemokine on leukemic cells, since they are not able to detect nor produce it.

Although there are many cells capable of secreting IL-8, monocytes and macrophages have an exacerbated capacity to produce this chemokine. In CLL patients, monocytes are not only increased in number in the circulation, but they are also recruited to the lymphoid tissues by the leukemic cells through the secretion of CCL3 and CCL4, once there they differentiate into Nurse-like cells (NLCs). In advanced stages of the disease, CD68+ myeloid cells (macrophage marker) number increases in the lymphoid tissues [9] as well as IL-8 plasmatic levels, suggesting that these cells constitute the main source of IL-8 in CLL patients.

Considering all data described before, we propose that a possible link between NLC-derived IL-8 and CLL progression could be TANs differentiation. This idea is in part supported by the fact that NETs-like structures were observed in close contact to leukemic cells in spleen biopsies of CLL-patients [10] and for our own results that suggest an association between a high percentage of circulating CD16^high^CD62L^dim^ neutrophils and advance disease. Even though some pieces of this puzzle are still missing, we encourage the CLL-research community to put NLC-IL-8-TAN axis under the spotlight by addressing the percentage of circulating CD16^high^CD62L^dim^ in peripheral blood samples, since its presence could give us an insight of what is happening in lymphoid tissues. In addition, linking it to several clinical variables in larger patient's cohorts could be helpful to confirm its value as a possible low-cost biomarker of CLL-progression (Figure 1).

**Figure 1:**
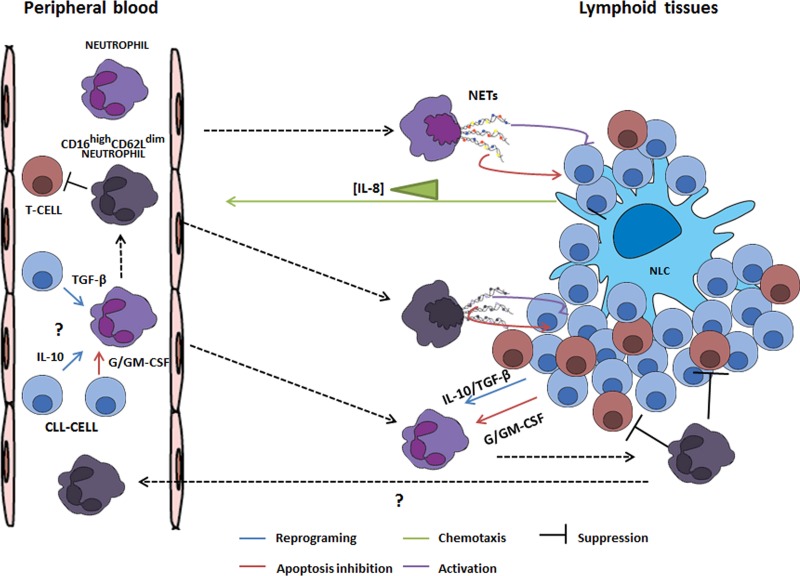
Schematic overview

## References

[1] Shaul ME (2018). FEBS J.

[2] Shen M (2014). PLoS One.

[3] Templeton AJ (2014). J Natl Cancer Inst.

[4] Podaza E (2017). Cancer Immunol Immunother.

[5] Podaza E (2019). Int J Cancer.

[6] Millrud CR (2017). Int J Cancer.

[7] Yoon JY (2012). Leuk Lymphoma.

[8] Risnik D (2017). Sci Rep.

[9] Jia L (2014). Blood.

[10] Sangaletti S (2014). Cancer Discov.

